# Supplementary figures and images for: Potential of MALDI-TOF MS biotyping to detect deltamethrin resistance in the dengue vector Aedes aegypti
Source: PLoS One. 2024 May 10;19(5):e0303027. doi: 10.1371/journal.pone.0303027 (PMC11086877; doi:10.1371/journal.pone.0303027)

## Slide 1
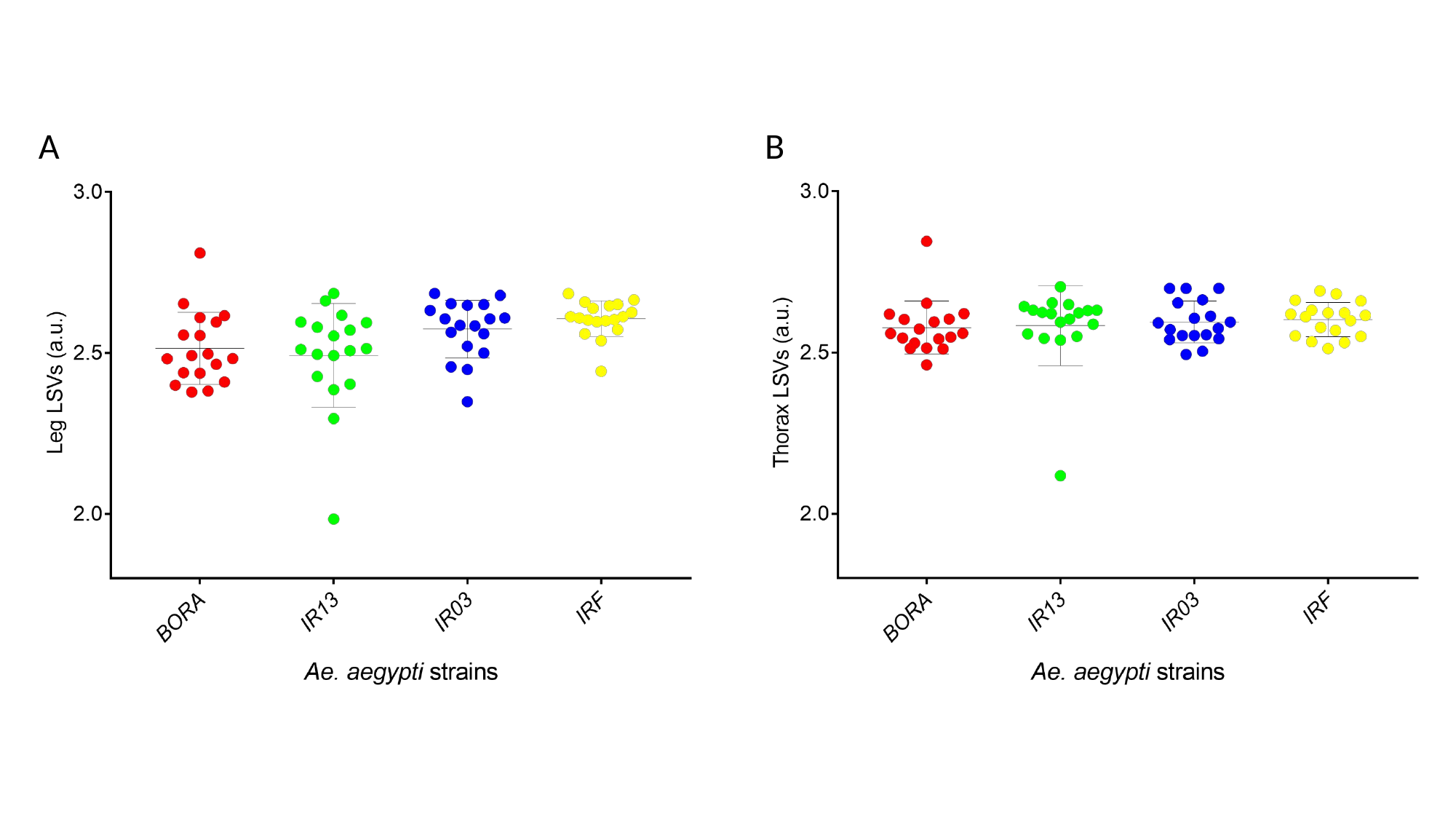

B
A

Supplement: S1 Fig — LSVs following upgrading homemade reference database with MS spectra from legs (A) and thoraxes (B) of the four Ae. aegypti lines. Eighteen specimens per line were tested. Horizontal dashed lines represent the threshold value for reliable identification (LSV>1.8). Red, green, blue and yellow dots correspond to BORA, IR13, IR03 and IRF Ae. aegypti lines, respectively. LSVs, log score values; a.u., arbitrary units. (PPTX) [file pone.0303027.s001.pptx]

## Slide 1
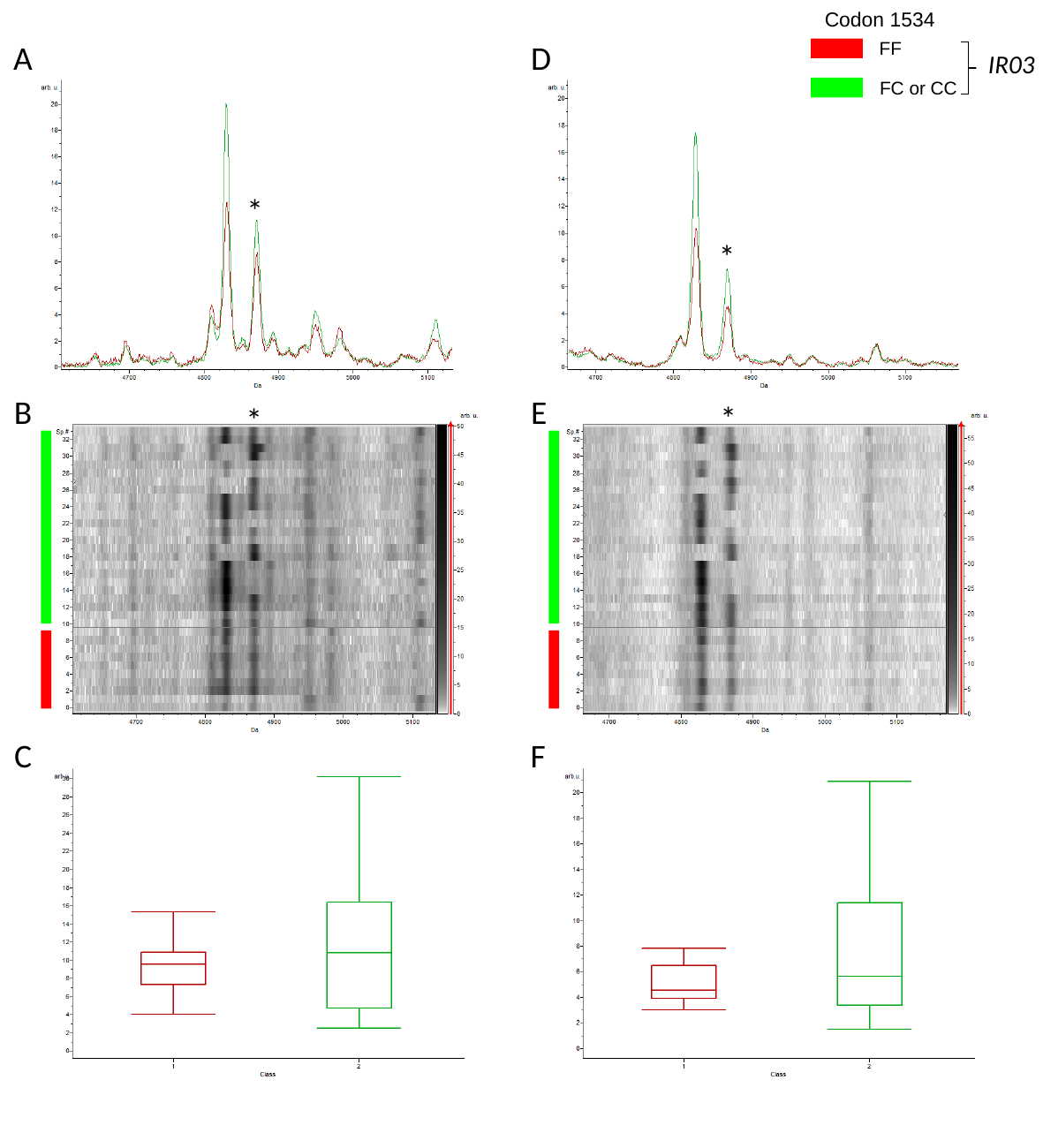

Codon 1534
A
D
FF
FC or CC
IR03
*
*
B
E
*
*
C
F

Supplement: S2 Fig — Overlay mean profile view of leg (A) and thorax (D) body parts according to 1534 genotype. Line color code of each genotype is indicated in the top right part. Gel view of leg (B) and thorax (E) MS spectra from the IR03 specimens per genotype. The two replicates loaded on the MS plate for each specimen per body part are presented. The discriminant MS peak (m/z: 4870 Da) is indicated by an asterisk (*). Graphical representation of the intensity of the 4870 m/z MS peak from legs (C) and thoraxes (F) according to 1534 genotype of IR03 Ae. aegypti line. Standard deviations of intensities are represented by vertical lines. A.U.: arbitrary units; m/z: mass to charge ratio; Red: haplotype homozygotes without mutation (VV/VV/FF); Green: mutant haplotype heterozygotes (VV/VV/FC) and homozygotes (VV/VV/CC). The same color code was used for all the panels. (PPTX) [file pone.0303027.s002.pptx]
