# Supplementary material for: Potential of MALDI-TOF MS biotyping to detect deltamethrin resistance in the dengue vector Aedes aegypti
Source: PLoS One. 2024 May 10;19(5):e0303027. doi: 10.1371/journal.pone.0303027 (PMC11086877; doi:10.1371/journal.pone.0303027)
Supplement: S1 Table — (DOCX) [file pone.0303027.s003.docx]

**Table S1.** Primer pairs used for detection of *kdr* mutation points.

| Primer name | Sequences | PCR product (bp) | Mutation points analyzed |
| --- | --- | --- | --- |
| 410F | TTACGATCAGCTGGACCGTG | 150 | V410L (GTA/TTA) |
| 410R | TTACGATCAGCTGGACCGTG |  |  |
| 1016F | ACAATGTGGATCGCTTCCC | 612 | V1016I (GTA/ATA) |
| 1016R | GCAATCTGGCTTGTTAACTTG |  |  |
| 1534F | TCGCGGGAGGTAAGTTATTG | 350 | F1534C (TTC/TGC) |
| 1534R | GTTGATGTGCGATGGAAATG |  |  |

F, forward; R, reverse.
