# Supplementary material for: Potential of MALDI-TOF MS biotyping to detect deltamethrin resistance in the dengue vector Aedes aegypti
Source: PLoS One. 2024 May 10;19(5):e0303027. doi: 10.1371/journal.pone.0303027 (PMC11086877; doi:10.1371/journal.pone.0303027)
Supplement: Attachment — Submitted filename: Responses_to_reviewer_Comments_R1.docx [file pone.0303027.s005.docx]

**Manuscript –**PNTD-D-23-01214

**Title:** Potential of MALDI-TOF MS biotyping to detect deltamethrin resistance in the dengue vector *Aedes aegypti*.

**Corresponding author:** Lionel ALMERAS.

**Journal:** Transfer from PLOS Neglected Tropical Diseases to *PLOS One*

**Editor comments:**

***General comment.***

**As with all papers reviewed by the journal, your manuscript was reviewed by members of the editorial board and by several independent reviewers. Based on the reviews, we regret that we will not be pursuing this manuscript for publication at PLOS Neglected Tropical Diseases.**

**I agree with the reviewers that the work doesn't offer much novelty, especially if the new peak of 4870 Da has not been characterized. However, although the data is preliminary for identifying deltamethrin-resistance mosquitoes, the work might be still of interest and therefore a transfer to PLOS One has been offered.**

***Responses to Editor comment’s***

Dear Editor,

We agree to submit the manuscript to Plos One.

Best wishes

The authors

**Reviewers comment’s**

***Summary and General Comments***

**Reviewer #1: The paper offers a detailed analysis of employing MALDI-TOF MS bio-typing to detect deltamethrin resistance in Aedes aegypti, a mosquito species infamous for spreading dengue. It critically assesses the shortcomings of current diagnostic methods and explores the potential of MALDI-TOF Mass Spectrometry in differentiating mosquito resistance to pyrethroids through protein profiles. The research reveals notable protein discrepancies, showcasing the method's potential for rapid and precise phenotype identification. Nevertheless, the technique requires additional verification for consistent insecticide resistance monitoring. Specifically, the absence of additional peptide de novo analysis to identify parent proteins raises concerns about the completeness and novelty of the study, leading to the recommendation of resubmission after providing substantiated proof of protein ID.**

**Reviewer #2: In this work, the authors exploited the already established MALDI-TOF-MS methodology to identify peptide signatures that will allow the identification of deltamethrin-resistant Aedes aegypti. The paper is methodologically sound and well executed. The problem I see is that the work is still preliminary for discriminating insecticide-susceptible vs resistant lines; there is a potential diagnostic ion at m/z 4870, which is present in several parts of resistant mosquitoes; however, this ion is also present in samples from susceptible mosquitoes and although it is present in lower intensity, its use as diagnostic is still premature. Therefore, given that this peak was not further characterized, the contribution of this work is only incremental respect to the work previously published by the same authors (Nebbak A and Almeras L 2020 Parasites & Vector).**

***Responses to reviewers comment’s***

*Both reviewers underlined that the main concern was the lack of information on the most discriminant MS peak, found independently in legs and thoraxes, at m/z 4870 allowing to distinguish Ae. aegypti resistant from susceptible colonies.*

*The identification of such peptide is actually not simple and requires complementary and highly sophisticate mass spectrometry apparatus. Moreover, the next step after peptide identification would be to validate its up-regulation in others Ae. aegypti deltamethrin-resistant specimens from different geographical areas which would take additional time. All these points were addressed in the discussion section of the manuscript (lines 372-376).*

*Once this peptide will be identified, it validation would be done either at the transcription (mRNA) or translation (polypeptides) levels. The quantification of it transcript, which is easier than protein detection, could be done by RT-qPCR. Nevertheless, as a delay exists between transcription and translation, the correlation between mRNA abundance and protein expression is not always linear. A validation, at the protein level by Western blot or ELISA, appears then more appropriate. Unfortunately the number of antibodies available against Ae. aegypti proteins is very limited which could hamper this validation. In such conditions, more complex and time-consuming label-free approaches would be necessary to develop like selected reaction monitoring (SRM), which allows an accurate identification of peptide of interest and it quantification (Kontostathi et al, Expert review of molecular diagnostics, 2019). This validation step is essential but requires too many resources and time to carry it out within tight deadlines, which is why the peptide identification and it validation will be the subject of a separate study.*

*It is interesting to remind the editor that the determination of mosquito life traits by MALDI-TOF MS profiling without identification of respective protein/peptide makers of interest didn’t impede the publication of results in high ranking journal. For example, Nabet et al. (Science Report, 2020) described the application of MALDI-TOF MS for determination mosquito age, blood feeding or Plasmodium infection. In this work, none of the MS peaks associated to these characters were selected for validation. Other studies investigating the efficiency of MALDI-TOF MS to distinguish mosquitoes infected or not by a pathogenic agents, like microfilariae (Tahir et al, Plos NTD, 2017) or murine Plasmodium (Laroche et al, Malaria Journal, 2017) didn’t identify the peaks detected by MALDI profiling as well.*

*In addition, the introduction of MALDI-TOF MS in microbiology, revolutionized micro-organism identification and it accuracy allowed to use it for routine diagnostic in microbiology laboratories. The micro-organism identification are uniquely based on their MS protein profiles matching with a reference database. The characterization of MS peaks is absolutely not necessary to validate MS identification. It is currently used for micro-organism identification but also for detection of antibiotic-resistances or to distinct bacteria strains. This method of MS peak comparison was repeatedly applied to differentiate sensitive from resistant bacteria of the same species (Pham et al, Journal of Antimicrobial Chemotherapy, 2020; Elbehiry et al, Diagnostics, 2023; Villarreal-Salazar et al, Indian J Med Microbiol, 2023), without characterizing peptides/proteins of interests. The detection of a protein signature related, directly or indirectly, to the resistance phenotype was sufficient to be used for sample classification.*

*Then, taken into account these previous studies, in the present work, we would like to pave the way of the use of MALDI-TOF MS profiling for the differentiation of susceptible from resistant mosquitoes to one pyrethroid insecticide. As underlined by the two reviewers, all the experiments were rigorously done and we paid attention to moderate the interpretation our results. Moreover, as proposed by the reviewers and as it was indicated initially in the manuscript, we underlined the necessity to better characterize this peptide in the near future.*

*We hope these answers correspond to reviewer expectations.*
