# Supplementary material for: Potential of MALDI-TOF MS biotyping to detect deltamethrin resistance in the dengue vector Aedes aegypti
Source: PLoS One. 2024 May 10;19(5):e0303027. doi: 10.1371/journal.pone.0303027 (PMC11086877; doi:10.1371/journal.pone.0303027)
Supplement: Attachment — Submitted filename: Responses_to_reviewer Comments_R1_PONE.docx [file pone.0303027.s006.docx]

**Manuscript -** Ref. No.: PONE-D-24-05959

**Title:** Potential of MALDI-TOF MS biotyping to detect deltamethrin resistance in the dengue vector *Aedes aegypti*.

**Corresponding author:** Lionel ALMERAS.

**Journal:** *PLoS ONE (ISSN 1932-6203)*

**Responses to reviewers’ comments**

**Line numbers correspond to the “tracked” version of the manuscript (folder name: *Main_text_Almeras_et_al_PONE_R1_tracking.pdf*), line number variations could occur between the “tracked” version and final version attributed to final file formatting.**

**Reviewer #1:**

***General comment.***

**The article "Potential of MALDI-TOF MS biotyping to detect deltamethrin resistance in the dengue vector Aedes aegypti" describes a new diagnostic approach based on protein profile signatures using MALDI-TOF-MS instrument and identification by an MS reference spectra database to determine pyrethroid insecticide resistance in mosquitoes. In this context, the study suggests performing a comparison of various MS profiles from four Ae. aegypti colonies, backed by statistical analysis tools. This audacious proposal seeks an alternative advanced diagnostic method using protein profiling data analysis by MALDI-TOF-MS to detect resistant mosquitoes at high-throughput levels.**

**The scientific work is really promising and crucial. Overall, the text is well written and abundant in experiment and outcome data. However, I believe the text is incomplete; certain decisive conclusions are not effectively interpreted and require more, pertinent interpretation. I agree to have this work published in the journal PLOS ONE only if the following adjustments are made and the issues are addressed.**

**Introduction, pages 3 to 4:**

***Comment 1.* Line 53: missing a reference**

***Response:*** *The reference [1] presented in the preceding sentence referred to points indicated in these two sentences. According to the reviewer, the reference was moved at the end of the sentence from* ***line 53****.*

***Comment 2.* Line 59: “…in vector control for decades has selected mosquito…” do you mean developed**

***Response:*** *This sentence indicated that the repetitive use of same chemicals has contributed to the selection of insecticide resistant mosquitoes worldwide. This phenomenon was observed for the large majority of the insecticides applied in the same area. Details about this point are available in the review [5] (****line 61****).*

***Comment 3.* Line 96: replace deltamethrin with pyrethroid ester insecticide.**

***Response:*** *As proposed by the reviewer, “pyrethroid deltamethrin” was replaced by “pyrethroid ester insecticide” (****line 97****).*

***Comment 4.*** **Sample homogenization and MALDI-TOF MS, pages 5-6 lines 137-150:**

**How did you perform the MALDI-TOF-MS calibration during the experiment? Please add the calibration method to the paper.**

***Response:*** *A total of four MALDI-TOF MS MicroFlex LT (Bruker) apparatus are available in our laboratory. These MALDI-TOF are used for routine microbiology diagnostic of human bacteria, yeast and fungi pathogens. To ensure the accuracy of the micro-organism identification results, each apparatus is controlled daily by one of the two specialist engineers based on the procedure described by the manufacturer. An automatic calibration of each instrument is done using a manufacturer-specified calibration standard, named Bruker Bacterial Test Standard (BTS, ref: #8255343). The BTS is an E.coli extract spiked with two high molecular weight proteins and has been developed for the Quality Control process of the MALDI Biotyper system. When BTS is used, MALDI Biotyper performs an automatic QC which includes calibration of the mass spectrometer, a laser setting check and evaluation of spectrum quality. System performance is finally confirmed by achieving E.coli identification which must pass minimum performance criteria.*

*In addition, legs or thoraxes from Ae. aegypti BORA specimens reared at the laboratory were loaded on each plate and were used as internal QC of sample preparation, notably for homogenization step, and of MALDI-TOF apparatus performance. The highly relevant and correct identification score (LSV >2.0) obtained for these both body parts in the present study (90% and 100% for legs and thoraxes, respectively) run against the reference database meets the manufacturer’s specifications. These identification parameters were used to validate each plate.*

***Lines 152-156:*** Daily, an automatic calibration of MALDI-TOF apparatus was done using a Bruker Bacterial Test Standard (BTS, ref: # 8255343). In addition, to control quality of mosquito homogenization step and MALDI-TOF apparatus performance of each plate, highly relevant and correct identification score (LSVs >2.0) should be obtained for at least more than half of the legs or thoraxes from *Ae. aegypti* BORA specimens queried against the reference MS database. These identification parameters were used as internal quality control to validate each plate.

**Database creation and blind tests pages 6 to 7 lines 173-183:**

***Comment 5.*** **-The quality of MALDI-TOF mass spectra may be affected by the following factors: technical knowledge of acquiring MALDI-TOF mass spectra, such as regular staff training and quality control of MALDI-TOF MS measurements, factors affecting spectrum acquisition settings, such as the number of laser shots applied and spectra averaged per measurement. Have you carried out routine diagnostics (QC tests) using standardized MALDI-TOF MS spectral quality?**

***Response:*** *The reviewer is right, several factors could perturb the quality of MALDI profiles. As indicated in the response to the previous comment from reviewer#1, all MALDI apparatus are daily calibrated, controlled and internal QC was loaded onto each plate. The high reproducibility of the spectra obtained among Ae. aegypti lines per body part from samples submitted to MS at successive days and sometime on distinct MALDI-TOF MS MicroFlex LT (Bruker) ensure a successful calibration and adequate performance of the MS apparatus. In addition, the correct identification species with relevant LSVs (>1.8 for 98.75% of spectra) obtained with MS spectra from both body parts queried against the DB are supplementary results supporting the correctness of the MALDI-TOF MS maintenance and performance.*

***Comment 6.* -Did you prepare any pooled sample by homogenizing both parts legs and thorax? This pooled sample could be applied as a quality control for MS data correction and normalization.**

***Response:*** *We thank the reviewer for this very pertinent comment. Effectively, the procurement of a sample which could be used as “a quality control for MS data correction and normalization” will improve the accuracy of the protein profiles comparisons. The mixing of legs and thoraxes could not be applied because resulting MS spectra correspond essentially to thorax MS profile due to the higher protein abundance in this body part compared to legs. The alternative could be to prepare a mix of few mosquito thoraxes or legs, separately. This kind of controls was already tested previously (unpublished data) and we observed that the intensity of MS spectra decreased with successive loading which could be attributed to repeated cycles of thaw and freezing. To avoid this phenomenon, aliquots were tested and we observed also a decreased of spectra intensity according to the duration of the storing of the aliquot in homogenization buffer. Moreover, we observed that protein profile intensity variations could occurred according to the efficiency of the sample homogenization step as previously reported (Bamou et al., Parasit Vectors 2022; Nebbak et al, Proteomics 2016). To limit this phenomenon, we decided to use a fresh or frozen mosquito which is treated in the same conditions as the other sample tested allowing to obtain a control of the quality of homogenization step and the buffer used for this sample destroying, as previously described (Costa et al., Trop Med Infect Dis 2023; Vega-Rua et al., Parasit Vectors 2018). This control is then treated in the same buffers and conditions as the other samples tested. In the present work, MS spectra from the Ae. aegypti BORA group were also used as controls (see response to the comment2 from reviewer#1 for details).*

**Results, pages 8-11 lines 195-296:**

***Comment 7.*** **-Lines 222-224: “These results highlighted that overall comparisons of MS profiles did not allow to clearly distinguishing specimens according to their deltamethrin resistance status whatever the body- part tested.” I do not agree with the statement above. In my opinion, your spectra will at least show the peak height intensity difference if you normalize them.**

***Response:*** *CCI and dendrogram are generated based on MSPs (Mean spectra profiles) corresponding to a representative spectra of sample replicates. During the process of CCI and dendrogram creations, the MSPs are normalized prior to be compared. Concerning PCA, it was made on single spectra (not MSPs), nevertheless an automatic normalization of spectra is done to success PCA analysis. Then, for all the analysis presented in the figure 2, a normalization of the spectra was firstly realized. To illustrate this normalization, a comparison of intensity of some selected peaks in legs or in thoraxes was done using ClinProTools (Supplementary figure A (see below), not include in the manuscript). The normalization of the MS spectra is illustrated by this list of peak selected presenting an equivalent intensity (ie, not significantly different) per body part independently of the mosquito line. Moreover, among the 99 and 118 MS peaks detected in the average spectra of legs and thoraxes, respectively, the intensity of solely 10 and 11 MS peaks were considered significantly different with FC > 1.3 between the deltamethrin resistant lines compared to the two susceptible lines. In the absence of normalization, it is probable that the intra-group heterogeneity could hamper the detection of these discriminant peaks. As spectra were already normalized, the results are then unchanged.*

***Comment 8.* -According to the PCA, there is a significant difference between BORA (French Polynesia) and the three others (French Guiana). I think you have multivariable factors to be taken into consideration.**

**-Have you tried multivariable study?**

**-Have you tried Partial Least-Squares Discriminant Analysis (PLS-DA)?**

***Response:*** *The reviewer is right, it is probable that multivariable factors could explain the difference between Ae. aegypti BORA and the three other isofemale lines. Nevertheless, the genetic background or geographic origin seems likely preponderant, notably based on previous works as underlined in the discussion section (****Lines 332-338****). All these analyses were performed using the soft provided by Bruker Daltonics for MALDI-TOF MS spectra analysis, including flexAnalysis v3.3, ClinProTools v2.2 and MALDI-Biotyper v3.0. (Bruker Daltonics). Unfortunately, neither PLS-DA nor others multivariable analyses are available with these software.*

***Comment 9.* -Line 263: “m/z of about 4870 Da”, I see in Table 1 that peak #16 is at m/z 4871.1 (TOF analyzer associated in general with high-resolution MS)**

**-Please add peak annotation (at least peptide sequence)**

**-Lines 253-257, Table 2 peak #29, I see m/z 4869.5 and not m/z 4870.**

**In my opinion, peaks #16 and #29 observed in both leg and thorax might be different**

***Response:*** *To confirm that the peaks #16 and #29 from leg and thorax are effectively the same peak, the MS spectra from the four lines and both body part were compared together using ClinProTools (Additional figure B (see below), not included in the manuscript). This comparison show unambiguously that the most discriminant MS peak found in leg (peak #16) and thorax (peak #29) profiles among the four Ae. aegypti lines was located at the same m/z position and was determined at m/z of 4870.5 Da. As the acquisition of the leg and thorax spectra was done in linear mode with m/z ranging from 2 to 20kDa, the window of peak resolution encompasses several Daltons and it is not surprising that small variations of peak position could occurred between spectra per body part if they are analyzed independently. As the start and end masses of the peak #16 and #29 were 4859.2 to 4883.3 and 4855.7 to 4883.0, respectively, it could be considered that these two peaks are the same and this peak was set at m/z of about 4870 Da. The peak resolution in MALDI profiling (linear mode) is insufficient to attribute relevant peptide sequence to a specific m/z position, which will be highly speculative. Spectra acquisition in reflectron mode is at least required but was not available on MALDI-TOF MS MicroFlex LT (Bruker) used in the present work. A sentence was added to support that peak #16 and #29 are identical.*

***Lines 246-249:*** As the start and end masses of the peak #16 (4871.1) and #29 (4869.5) were at m/z from 4859.2 to 4883.3 and from 4855.7 to 4883.0, respectively, it was considered that these two peaks are the same. The m/z of this discriminant peak found in both body parts was set at about 4870 Da.

***Comment 10.* -Page 11, lines 278-296 Potential association between the 4870 m/z MS peak and kdr mutations: Indeed, this is a very important study; did you consider associating other m/z significant MS peaks? Why only 4870 m/z? the other m/z values have a fold change of more than one.**

**-The discriminant MS peaks (observed in Tables 1&2 form legs and thorax deltamethrin-resistant (IR03 and IRF) and susceptible (BORA and IR13) Ae. aegypti line (BORA and IR13) Ae. aegypti lines) are all significant and they all present a potential biomarker for the resistant-group species. In this case, you should reconsider the data interpretation to value more these findings.**

***Response:*** *The research of a potential association between discriminant peaks and kdr mutations was firstly investigated for 4870 m/z MS peak, because it was detected in both body parts and was the most up-regulated discriminant peak in deltamethrin-resistant lines. Unfortunately, among the three sites (V410L, V1016G/I, and F1534C) of the kdr mutations genotyped, changes were noticed in two third of the specimens from only one line (IR03). In such condition, it was not possible to associate a particular genotype to a specific phenotype which include at least two mosquito lines. Then, the research of potential association with other discriminant peak appears not realistic. No particular change was done concerning this point.*

**Discussion**

***Comment 11.*** **Line 361, the discussion should be extended to other m/z ions observed in table 1 and 2**

***Response:*** *The reviewer is right that the peak at m/z 4870 focused our attention. In addition to the criteria evoked above for this peak, compared to the remaining MS peak in legs and thoraxes, the 4870 peak is among the top one for legs and top two for thoraxes if we take into account average peak intensity. Moreover, if we take into account their intensity in the whole profile, they found among the more abundant as indicated in the response to comment #3 from reviewer #2. As in this section, it was discussed the difficulty to characterize peptide sequence of respective MS peaks, the abundance of peak of interest is an essential parameter. Then, this point was underlined in the result and discussion section, as follow:*

***Lines 251-254:*** Moreover, with a respective average peak intensity of 11.1 arbitrary units (a.u.) and 6.7 a.u. for the leg and thorax peaks at about m/z 4870, these peaks were classified in the top ten and top thirty for leg and thorax profiles from deltamethrin-resistant lines (IR03 ad IRF).

***Lines 373-377:*** Although the identification of the other discriminant peaks could be helpful to establish their involvement in phenotypic deltamethrin-resistant trait, their lower peak intensity could compromise these characterizations. Conversely, the peak at m/z 4870 was among the top one for legs and top two for thoraxes of discriminant average peak intensity. Moreover, taking into account the whole spectra, this peak was among the more abundant.

***Comment 12.*** **Conclusion: the conclusion is very general and brief**

**-Rewrite the conclusion to highlight the importance of MALDI-TOF-MS in monitoring deltamethrin resistance in the dengue vector Aedes aegypti. I agree that this pioneering effort requires further development in the future. However, you should put more value to the findings in this work especially related to the m/z list found in Tables 1 and 2, which in my opinion should be more discussed and analyzed pertinently. This work is missing a biological survey of the other peak discovered, which would provide us with a better understanding of the true biomarker associated with deltamethrin resistance.**

***Response:*** *The conclusion already mentions the importance of this approach in the analysis of “*mosquito life traits*” which “*will be revolutionary for vector biology and the prevention of mosquito-borne diseases outbreaks*”. Concerning the discussion about the identification of the other discriminant peaks found in leg and thorax spectra, this point was already mentioned in the responses to comment 10 and to comment 11 from reviewer #1. Moreover, as indicated in the response to comment 3 from reviewer #2, the m/z peak at about 4870 kDa is among the most intense discriminant peaks and also the well detectable among the whole protein leg and thorax profiles. In such conditions, a priority to this particular peak should be privileged. Then, no particular change was done concerning this point.*

**Figures**

***Comment 13.*** **-Figure 1: The mass spectra have different intensity scales; please consider normalizing the MS by putting a common intensity scale for better comparison.**

***Response:*** *In the Figure 1 are presented two spectra from each line from both body parts, analyzed with Flexanalysis software (Bruker). The aim of this figure was to show representative spectra for which intensity variations could occurred among MS profiles. This software did not allow to perform spectra normalization which could be done only with ClinProTools v2.2 or MALDI-Biotyper v3.0. (Bruker Daltonics), as indicated in the response to comment #7 from reviewer #1. Then, it is not possible to normalize spectra on this figure with Flexanalysis software.*

***Comment 14.* -Figure 2: PCA is not clear and the legend is very small, please reconsider the font size of the figure to be better readable.**

***Response:*** *We recognize that the quality of the PCA need to be improve. They are generated automatically by ClinProTools v2.2 software (Bruker Daltonics) and uniquely copy and paste of images was possible which explain the low quality of the panels. To improve the reading of the PCAs and their legend, they were enlarged and directly included in the new Figure 2. The yellow dots are also not well readable, however the selection of the colors is not possible in this software.*

***Comment 15.* -Figure 3 (A &D) is not clear, it is difficult to read the m/z for the peak you mentioned.**

***Response:*** *All the images presented in the Figure 3 were copy and paste from ClinProTools v2.2 software (Bruker Daltonics). To improve reading, all the numbers on “x” and “y” axis were manually rewritten. A new Figure 3 is now available.*

**Reviewer #2:**

***General comment.* In this paper, the authors have utilised MALDI-TOF-MS protein/peptide fingerprinting in an attempt to find any discriminating peaks between different Ae. aegypti deltamethrin resistant and susceptible lines. The analysis was performed using two body parts, thorax and legs. The resulting spectra was compared and analysed using various statistical tools leading to the identification of a peak at m/z 4870 representing the higher fold change (>2.8 fold) between resistant and susceptible-lines for both body parts.**

**I have some points that need to be clarified regarding the MS data analysis and the two peaks at m/z 4871.1 and m/z 4869.5 detected from legs and thorax, respectively.**

***Comment 1.* The difference between m/z 4871.1 and m/z 4869.5 is 1.6 Da. This indicates that these two peaks could be completely different as most peptide searches are usually done within +/-0.2 Da. So, what are the criteria used to decide if two peaks are similar or not?**

***Response:*** *We thank the reviewer for this very interesting remark. This point was already mentioned by the reviewer #1, then we proposed to see the responses done to the comment 9 from reviewer#1.*

***Comment 2.* Also, it is not clear in the manuscript what mass tolerance used for searching the spectra against the reference MS spectra from the 16 distinct mosquito species. Is this database available for everyone to use and approved for reliable species identification? Is it just a list of m/z values? Does it include Protein/Peptide IDs? Were the IDs supported by tandem mass spectrometry (MS/MS)?**

***Response:*** *Spectral matching of the query spectra against the MS reference spectra DB was done with MALDI-Biotyper v3.0. (Bruker Daltonics) with default setting parameters (i.e., parameters used for bacteria identification). The mass tolerance of adjusted spectrum and for a peak were set at 250 ppm and 600 ppm, respectively. Concerning the 16 distinct mosquito species used as reference spectra included in the DB, they are all available by everyone because these reference MS spectra come from two distinct study which provided free access to these raw MS spectra (Vega-Rua et al, Parasit Vectors 2018; Briolant et al, PloS One 2020). A validation of specimen identity by gene sequencing was done for all respective spectra included in the reference DB. Moreover, the reliability of these spectra for species identification was validated in another previous study (Costa et al, Trop Med Infect Dis. 2023). These reference MS spectra correspond to a list of m/z values plus their respective peak intensity. The identity of each peak was totally unknown which is the principle of MALDI-TOF MS profiling for which the generation of specific patterns (i.e., mass fingerprint) allows to classify a sample. No particular change was done concerning this point.*

***Comment 3.* These two peaks (m/z 4871.1 and m/z 4869.5) have extremely low signal intensities in the range of 2-11 a.u (Tables 1 and 2). So, what are the parameters used to evaluate the data and/or to filter real signals from noise? i.e. what is the minimum S/N ratio, signal intensity or relative intensity to create a peak list of 99 and 118 peaks detected in legs and thoraxes, respectively?**

***Response:*** *We thank the reviewer for this interesting comment. The values of average peak intensity are indicated in arbitrary units. In the leg spectra, independently of the mosquito line, the most intense peak have an average peak intensity of 27.5 a.u.. With an average intensity of 11.1 a.u. for the peaks at m/z 4871.1, this peak is classified in the top ten for leg profiles from deltamethrin-resistant lines (IR03 ad IRF). In the thorax spectra, the most intense peak have an average intensity of 49.2 a.u.. With an average intensity of 6.7 a.u. for the peaks at m/z 4869.5, this peak is classified in the top thirty for thorax profiles from deltamethrin-resistant lines (IR03 ad IRF). Then, these two peaks could not then considered to present an “extremely low signal intensities”. As indicated in the manuscript, default parameters were used for spectra analysis with the different software (ie,* ***line 168:*** “… MALDI-Biotyper v3.0. software with default settings …” ; ***line 171:*** “…ClinProTools v2.2 software was used with the default settings.”). *For example, the signal to noise threshold was set at 5.00, the maximum number of peak detected is 100, the peak aggregation width was set at 500 ppm and intensity was used for peak calculation for ClinProTools v2.2 software. As default parameters were used and were already indicated in the manuscript, no change was done concerning this point.*

**Journal Requirements:**

**When submitting your revision, we need you to address these additional requirements.**

**1. Please ensure that your manuscript meets PLOS ONE's style requirements, including those for file naming. The PLOS ONE style templates can be found at**

[**https://journals.plos.org/plosone/s/file?id=wjVg/PLOSOne_formatting_sample_main_body.pdf**](https://journals.plos.org/plosone/s/file?id=wjVg/PLOSOne_formatting_sample_main_body.pdf) **and** 
[**https://journals.plos.org/plosone/s/file?id=ba62/PLOSOne_formatting_sample_title_authors_affiliations.pdf**](https://journals.plos.org/plosone/s/file?id=ba62/PLOSOne_formatting_sample_title_authors_affiliations.pdf)

***Response:*** *Done*

**2. We note that the grant information you provided in the ‘Funding Information’ and ‘Financial Disclosure’ sections do not match.**

**When you resubmit, please ensure that you provide the correct grant numbers for the awards you received for your study in the ‘Funding Information’ section.**

***Response:*** *Correction done.*

**3. Thank you for stating the following financial disclosure:**

**"AL received the following award
This work has been supported by the Délégation Générale pour l’Armement (DGA), MSProfileR project, Grant no PDH-2-NRBC-2-B-2201
This work was also supported by the WIN (Worldwide Insecticide resistance Network)"**

**Please state what role the funders took in the study.  If the funders had no role, please state: "The funders had no role in study design, data collection and analysis, decision to publish, or preparation of the manuscript." If this statement is not correct you must amend it as needed.**

**Please include this amended Role of Funder statement in your cover letter; we will change the online submission form on your behalf.**

***Response:*** *All these points were taken into account and included in the cover letter.*

**Cover letter:** According to the journal comments, we state that the funders had no role in study design, data collection and analysis, decision to publish, or preparation of the manuscript.

**4. Thank you for stating the following in the Acknowledgments Section of your manuscript:**

**"We thank the “Fondation Méditerranée Infection (FMI)” which offered personnel grant to MMC and the WIN (Worldwide Insecticide resistance Network) which contributed in the travelling of the student between Brazil and France."**

**Please note that funding information should not appear in the Acknowledgments section or other areas of your manuscript. We will only publish funding information present in the Funding Statement section of the online submission form.**

**Please remove any funding-related text from the manuscript.**

***Response:*** *The funding information were corrected according to guidelines.*

***Lines 422-424:* Acknowledgments.** We thank the “Fondation Méditerranée Infection (FMI)” and the WIN (Worldwide Insecticide resistance Network) for their administrative support.

**5. We are unable to open your Supporting Information file [Additional_file_1_DB.7z]. Please kindly revise as necessary and re-upload.**

***Response:*** *The “additional file 1” was saved in another file format and re-uploaded (.zip file).*
